# Spectrum of Morphological and Immunofluorescence Patterns in Lupus Nephritis: A Single Institutional Study

**DOI:** 10.7759/cureus.25363

**Published:** 2022-05-26

**Authors:** Saira Javeed, Safana Sadaf, Saima Batool, Ayma Batool, Zubaria Rafique, Akhtar S Chughtai

**Affiliations:** 1 Histopathology, Chughtai Institute of Pathology, Lahore, PAK

**Keywords:** anti-dsdna antibody, immunofluorescence, end stage renal disease, systemic lupus erythematosus, lupus nephritis

## Abstract

Introduction

Lupus nephritis (LN) is a systemic manifestation of systemic lupus erythematosus (SLE). LN commonly occurs three to five years later after the onset of SLE and is one of the leading cause of end-stage renal disease. The objective of this study was to evaluate the spectrum of morphological and immunofluorescence (IF) patterns in LN.

Methodology

A cross-sectional descriptive study was conducted on 58 renal core biopsies diagnosed as LN at Chughtai Institute of Pathology between January 2021 and December 2021. Based on the International Society of Nephrology and the Renal Pathology Society, prevalence of different classes of LN was assessed. The demographic, clinical, and biochemical parameters were analyzed in association with different histological classes of LN.

Results

In our study, the male-to-female ratio was 1:6.5. The mean age was 23.09 ± 9.23 years. Increased serum urea levels were found in 36 (62.10%) patients, and increased serum creatinine levels were found in 43 (74.12%) patients. Nephritic range proteinuria was seen in 14 (24.10%) patients, while 44 (75.90%) patients had proteinuria in the nephrotic range. Anti-double stranded DNA antibody was positive in 49 (84.50%) patients. Microscopic hematuria was present in 46 (79.30%) patients. Main bulk of patients belong to class V, 25 (43.10%), followed by class IV, 16 (27.59%). Full-house IF pattern was seen in majority of patients.

Conclusion

This study showed a high frequency of prevalence of advanced classes of LN, i.e., class V followed by class IV. There is a strong diagnostic utility of IF in LN. Similarly, full-house IF pattern was observed in majority of patients in our study, irrespective of which class of LN they belonged to.

## Introduction

Systemic lupus erythematosus (SLE) is an autoimmune disorder with a high risk of morbidity and mortality as it results in many complications. Lupus nephritis (LN) is renal manifestation and common complication of SLE. LN commonly occurs three to five years later after the onset of SLE and is one of the leading causes of end-stage kidney disease [1]. Highest incidence of SLE is observed in North America. In Pakistan, its prevalence is half-way between Caucasians and other Asians [2]. It is mainly a disease of adults, with preponderance in women of reproductive age. There is a strong genetic predisposition observed in SLE, most commonly in human leukocyte antigen (HLA) upon exposure to environmental factors [3].

The word “lupus” belongs to the Latin language that means “wolf.” it is named so because the patients of SLE have a specific type of rash over their face that resembles a wolf bite. The first case of LN was diagnosed by Hippocrates, who is known as the “father of medicine” [1]. Diagnosis of SLE is made on the basis of clinical picture and serology. The clinical appearance has a widespread variety as it is a multiorgan disorder. Renal involvement is initially determined by routine laboratory tests that include urine analysis and renal function tests [4]. Proteinuria is observed in almost every case and gives warning to assess kidney status through renal biopsy [5]. Microscopic hematuria is also almost always present. There are no well-defined criteria regarding when to perform renal biopsy, but different studies recommended to perform kidney biopsy if proteinuria is >500mg/dL in the presence or absence of clinical symptoms or if renal function tests such as serum creatinine and estimated glomerular filtration rate are impaired with any level of hematuria and proteinuria in the absence of any other cause. Apart from anti-double stranded DNA (anti-dsDNA) antibodies, other factors such as antinuclear antibody (ANA) and decreased complement levels for C3 and C4 also help reach the diagnosis, but since the clinical picture in many cases underestimates the frequency of disease, histological evidence is very important as it also aids in deciding treatment [6,7].

The International Society of Nephrology (ISN) and the Renal Pathology Society (RPS) have categorized LN into six classes on the basis of histological evaluation of changes seen in renal structures in all four compartments, glomeruli, tubules, Interstitium, and blood vessels, as a result of different responses of immune process in different patients [6,8]. In LN, immune complex deposits, immunoglobulin G (IgG), immunoglobulin A (IgA), immunoglobulin M (IgM), and complement (C1q and C3) are also present, which are determined by immunofluorescence (IF). The locations of these deposits are different in different classes, which help in diagnosis. Subclassification is done in LN because there is marked variability for treatment protocol for each class. Early diagnosis helps in better outcomes.

In the present study, we aimed to evaluate the spectrum of morphology, IF patterns, and the percentage of patients in different classes of LN.

## Materials and methods

This is a cross-sectional descriptive study conducted after approval from the Institutional Review Board (IRB) Committee of Chughtai Institute of Pathology, Lahore, Pakistan (Reference letter no. CIP/IRB/1055). All the renal biopsies diagnosed as LN over a period of one year between January 2021 and December 2021 with complete clinical and serological data were included in the study. Cases were extracted from archives of Chughtai’s lab using the Nexus software. Patients lacking relevant serological information that includes serum creatinine, serum urea, C3 and C4 levels, ANAs, anti-dsDNA antibodies, and all renal biopsies with suspected LN clinically and morphologically but with non-contributing IF findings were excluded from the study. Hence, the study included a total of 58 cases collected through a convenient sampling technique.

**Figure 1:**
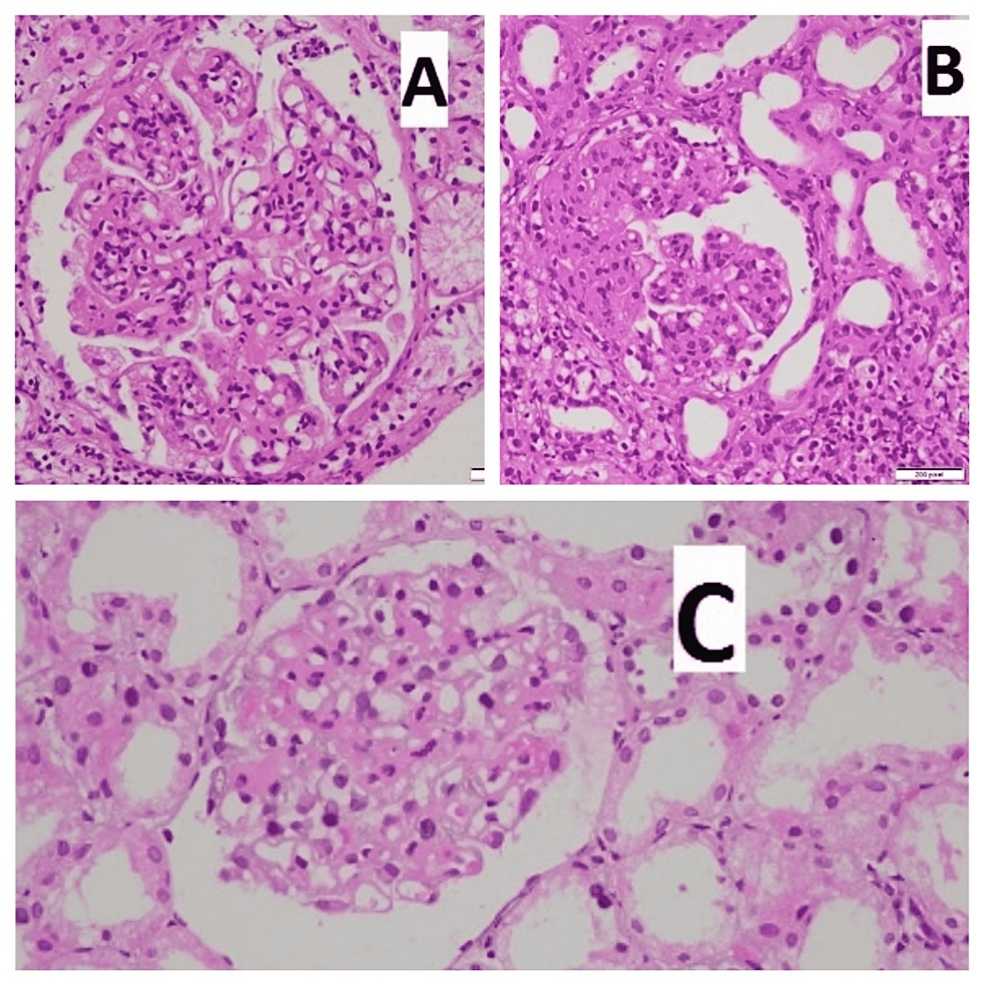
(A) Glomeruli in class III lupus nephritis showing mesangial hypercellularity (H&E x400) (B). Glomeruli in class IV lupus nephritis showing cellular crescent (H&E x400) (C). Glomeruli in class V lupus nephritis showing membranous thickening (H&E x400). H&E, hematoxylin and eosin

**Figure 2:**
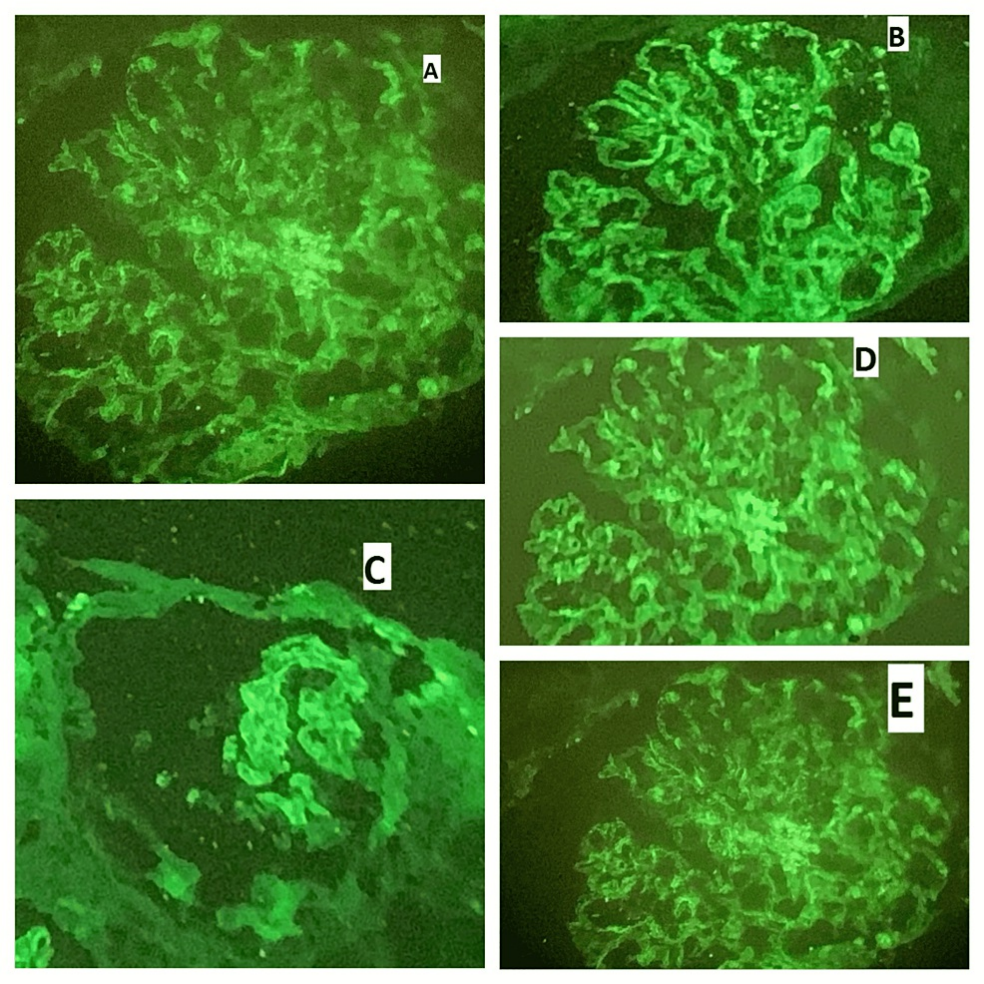
Full-house immunofluorescence in lupus nephritis showing (A) granular pattern of IF for IgG (400x), (B) granular pattern of IF for IgM (400x), (C) granular pattern of IF for IgA (400x), (D) granular pattern of IF for C3 (400x), and (E) granular pattern of IF for C1q (400x). IF, immunofluorescence

Two samples of each renal biopsy specimen were received: one was used for light microscope (LM) and the other for IF study. All biopsies were processed for both light and IF microscopy as per the standard protocols followed at our center, i.e., the core for light microscopy was preserved in 10% buffered formalin, and for IF it was preserved in normal saline. For light microscopy, core was sectioned at 2-4 µ thickness and stained with hematoxylin and eosin (Figure 1). Special stains such as periodic acid-Schiff (PAS), Gomori methenamine silver (GMS), and Masson’s Trichrome stains (BioGnost, Zagreb, Crotia) were also performed. The core for IF was frozen sectioned at 3-4 µ on cryostat at -20°C. IF in our institute was done by direct method. Antibodies against IgG, IgM, IgA, C3, and C1q (all from Dako, Glostrup, Denmark) were used and then interpreted in terms of intensity, pattern, and type of fluorescence (Figure 2). Clinicopathological correlation was attempted. Diagnosis was made on the basis of LM and IF only as electron microscopy was not available in our institute. Biopsies showing more than or equal to 10 glomeruli were considered adequate for diagnosis. All cases were reviewed by two consultant renal pathologists with complete agreement. The numerical variables such as age, serum urea, and serum creatinine were analyzed as mean and standard deviation. The categorical variables such as gender, proteinuria, haematuria, C3 levels, C4 levels, ANA, anti-dsDNA, diagnosis of different classes of LN, and IF expression were calculated as frequency and percentages.

Data were analyzed, and statistical analysis was performed using the Statistical Package for Social Sciences (SPSS) software, Version 22 (IBM Corp., Armonk, NY, USA). Fisher’s exact test was applied wherever applicable. A p-value of less than 0.05 was considered as statistically significant.

## Results

In our study, a total of 58 cases were included, out of whom 8 (13.80%) were male and 50 (86.20%) were female. The age range was 3 to 50 years, with a mean age of 23.09 ± 9.23 years. Out of 58 patients, 36 (62.07%) patients were below the age of 25. Serum urea levels ranged from 12 mg/dL to 107 mg/dL (normal = 10-45 mg/dL) with mean serum urea level of 56 ± 22.66 mg/dL. Serum urea levels were found to be disturbed in 36 (62.10%) patients. Serum creatinine levels ranged from 0.40 mg/dL to 13.00 mg/dL (normal = 0.6-1.11 mg/dL). Abnormal serum creatinine levels were noted in 43 (74.13%) patients, with mean serum creatinine level of 3.7 ± 3.29 mg/dL. Majority of patients in our study showed proteinuria and microscopic hematuria in either the nephrotic range (>3.5 g/24 hours) or nephritic range (<3.5 g/24 hours). Out of 58 patients, 14 (24.10%) patients had proteinuria in the nephritic range and 44 (75.90%) patients had proteinuria in the nephrotic range. Microscopic hematuria was present in 46 (79.30%) patients. ANA was found to be positive in 52 (89.70%) patients, while anti-dsDNA was positive in 49 (84.50%) patients. Serum C3 levels were decreased in 50 (86.20%) patients, while serum C4 levels were decreased in 51 (87.90%) patients. All the basic clinical and laboratory features of patients at the time of biopsy are given in Table 1.

**Table 1:** Demographic, clinical, biochemical, and laboratory parameters of patients with lupus nephritis (n=58) ANA, nuclear antibodies; anti-dsDNA, anti-double stranded DNA

Demographic, Clinical, Biochemical, and Laboratory Parameters	n (%)
Male	8 (13.80%)
Female	50 (86.20%)
Positive ANA	52 (89.70%)
Positive anti-dsDNA	49 (84.50%)
Decreased C3	50 (86.20%)
Decreased C4	51 (87.90%)
Hematuria - present	46 (79.30%)
Hematuria - absent	12 (20.70%)
Protein urea - nephrotic range	44 (75.90%)
Protein urea - nephritic range	14 (24.10%)

**Figure 3:**
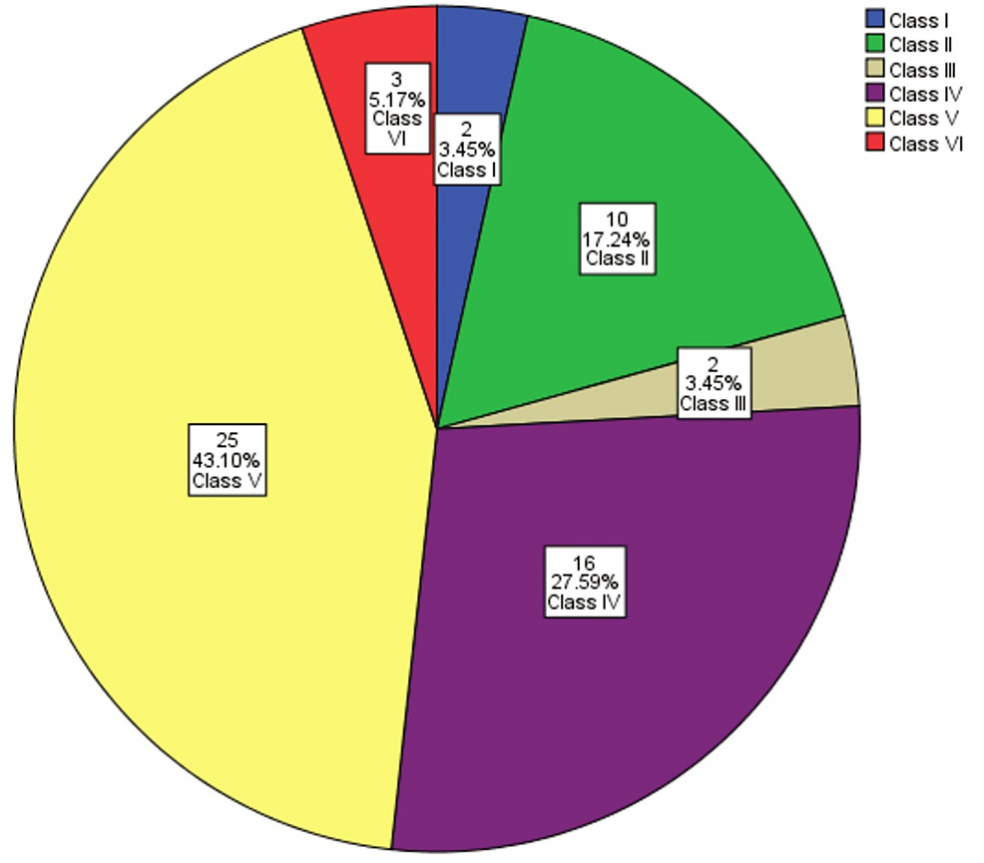
Frequency of distribution of patients in different classes of lupus nephritis on the basis of histopathology (n=58).

The main bulk of patients belong to class V, 25 (43.10%), followed by class IV, 16 (27.59%) (Figure 3).

**Figure 4:**
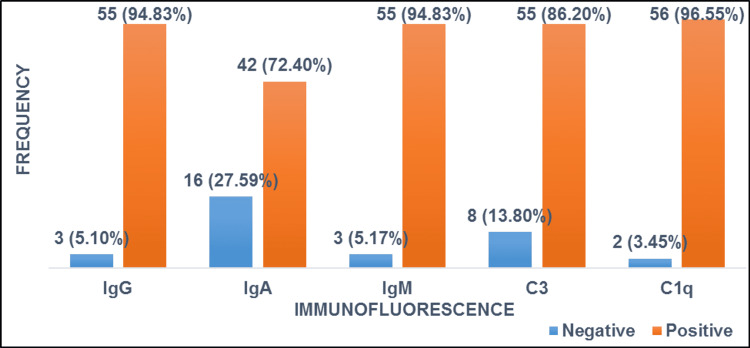
Frequency of immunofluorescence expression in lupus nephritis (n=58).

Among all IF antibodies, maximum positivity of C1q was observed, which turned out to be positive in 56 (96.55%) patients, followed by equal positivity for C3 and IgG in 55 (94.83%) patients. The frequency of IF expression (Figure 4) and frequency of IF expression in relation to different classes of LN, which was statically significant, can be seen in Table 2. Clinical, biochemical, laboratory, and histopathological connotation among different classes of LN are given in Table 3.

**Table 2:** IF pattern in lupus nephritis according to different classes (n=58) IF, immunofluorescence; Ig, immunoglobulin

IF	Class I n=2	Class II (n=10)	Class III (n=2)	Class IV (n=16)	Class V (n=25)	Class VI (n=3)	Total (n=58)	p-Value
IgG	1 (50.00%)	8 (80.00%)	2 (100.00%)	16 (100.00%)	25 (100.00%)	3 (100.00%)	55 (94.83%)	0.058
IgA	0 (0.00%)	4 (40.00%)	2 (100.00%)	15 (93.75%)	19 (76.00%)	2 (66.67%)	42 (72.41%)	0.002
IgM	1 (50.00%)	9 (90.00%)	2 (100.00%)	15 (93.75%)	25 (100.00%)	3 (100.00%)	55 (94.83%)	0.073
C3	0 (0.00%)	8 (80.00%)	1 (50.00%)	14 (87.50%)	24 (96.00%)	3 (100.00%)	50 (86.20%)	0.003
C1q	1 (50.00%)	9 (90.00%)	2 (100.00%)	16 (100.00%)	25 (100.00%)	3 (100.00%)	56 (96.55%)	0.023

**Table 3:** Clinical, biochemical, laboratory, and histopathological connotation among different classes of lupus nephritis (n=58)

		Class I (n=2)	Class II (n=3)	Class III (n=4)	Class IV (n=16)	Class V (n=25)	Class VI (n=3)	Total n=58	p-Value
Gender	Male	1	2	1	0	3	1	8	0.05
	Female	1	8	1	16	22	2	50	
Age group	≤25 years	1	7	2	13	13	0	36	0.05
	>25 years	1	3	0	3	12	3	22	
Serum urea	≤45 mg/dL	2	7	0	6	7	0	22	0.03
	>45 mg/dL	0	3	2	10	18	3	36	
Serum creatinine	≤1.1 mg/dL	1	5	0	4	4	1	15	0.27
	>1.1 mg/dL	1	5	2	12	21	2	43	
Hematuria	Absent	2	7	0	2	0	1	12	<0.001
	Present	0	3	2	14	25	2	46	
Proteinuria	≤3.5 g/24 hour	2	9	0	2	0	1	14	<0.001
	>3.5 g/24 hour	0	1	2	14	25	2	44	
Active lesions	Absent	2	10	0	0	25	3	40	<0.001
	Present	0	0	2	16	0	0	18	
Chronic lesions	Absent	2	10	0	0	25	3	40	<0.001
	Present	0	0	2	16	0	0	18	

## Discussion

LN is a frequent and dire manifestation of renal involvement in patients of SLE. In majority of cases of SLE, histological involvement of kidneys is evident even in the absence of clinical presentation. The sole purpose of this study was to evaluate the spectrum of morphology, IF patterns, and the proportion of patients in different classes of LN. In our study, we found that majority of patients were female, 50 (86.20%). The male-to-female ratio was 1:6.5 in our study, which is similar to other studies on LN, such as the studies by Hashmi et al. and Farah et al. [5,9]. C3 and C4 levels were decreased in 50 (86.20%) and 51 (87.90%) patients, respectively. These clinical findings are similar to a study performed in a Jordanian tertiary medical centre by Farah et al. in which they found that C3 levels were decreased in 75.90% patients and C4 levels were decreased in 44.30% [9]. ANA was found positive in 52 (89.70%) patients. Anti-dsDNA antibody was found positive in 49 (84.50%) patients. This percentage is nearly similar to those reported by Goilav and Putterman in their study of role of these antibodies in pathogenesis of LN [10]. The presence of anti-dsDNA antibody indicates the background of systemic lupus erythematous with renal involvement. The prevalence rate of LN is different in different regions of the world [11].

We present a study in which the highest frequency was found of class V LN, 25 (43.10%), followed by class IV, 16 (27.58%), which is different from that seen in different studies conducted worldwide and in Pakistan in which the highest prevalence of class IV followed by class V was noted [5,9]. The more frequent prevalence of class V in our study may be due to the fact that we receive renal biopsies from all over Pakistan including the backward regions of our country where patients most of the time present at advanced stages because of lack of access to health care and poor infrastructure. Minimum number of cases in our study were of class I LN, 2 (3.45%), followed by class VI, 3 (5.17%). The most precise justification for a smaller number of cases of class I could be explained in terms of no obvious clinical indication and abnormal laboratory profile early in the disease course and thus renal biopsies are usually not performed at this stage in our region [12]. Less number of cases of class VI may be due to end-stage renal disease in which >90% of glomeruli are sclerosed and thus clinicians mostly do not prefer renal biopsy at this stage.

Full-house IF pattern was seen in majority of patients irrespective of which class of LN they belonged to. These results correlate with the study by Singh et al. in which they observed 100% diagnostic utility of IF in LN [13].

Active and chronic lesions were assessed only in 18 (31.03%) cases, which belong to class III and class IV LN. This feature conforms to majority of studies conducted previously worldwide that active and chronic lesions are mostly present in class III and class IV [14]. In active lesions, glomerular endocapillary proliferation was noted in 17 (94.44%) patients, while wire loop deposits were seen in 16 (88.89%) patients. In chronic lesions, maximum frequency (100%) was observed for tubular atrophy and interstitial fibrosis. These features are clinically important as different studies have correlated the clinical renal outcome other than histological classes of LN with the features of activity and chronicity [15]. Also, tubulointerstitial damage is a strong predictor of poor survival in long-term LN [16].

The histological classification of various classes of LN is important in management as well as in prognosis. This is because each class has different treatment protocols, and treatment strategies have evolved over a period of time from incurable to improved outcomes [17,18]. In addition to those, early classes of LN responds well to medical treatment.

Limitations

Our study has a few limitations that include small sample size and lack of clinical follow-up to see progression of one class to other or to end-stage kidney disease or remission of disease. Hence, we also endorse the suggestion of Hoover and Costenbader and Morales et al. that those patients diagnosed with LN should be evaluated clinically after every three to six months with laboratory tests for proteinuria and hematuria to assess the renal status to see renal response to treatment followed by renal biopsy at regular interval for sustaining the diagnosis of LN [18,19]. Serious protocols for evaluation, diagnosis, and follow-up are strongly suggested as instead of latest advances, LN still have high morbidity and mortality and leads to end-stage kidney disease [20].

Also, as this was a single-center study, the results may not reflect the true prevalence of different classes of LN in our country. Furthermore, lack of electron microscopy at our institute precludes further assessment.

## Conclusions

In a nutshell, our study highlighted that a large number of cases of LN fall in advanced classes according to the international renal classification, i.e., class V followed by class IV. There is a strong diagnostic utility of IF in LN. Similarly, in our study, full-house IF pattern was seen in majority of patients irrespective of which class of LN they belonged to. Maximum utility of C1q followed by C3 and IgG was also observed.
